# Prolonged Fever in a Multidrug-Resistant Typhoid Fever Patient Despite Appropriate Antimicrobial Therapy: A Case Report

**DOI:** 10.7759/cureus.78999

**Published:** 2025-02-14

**Authors:** Abdulrahiman Cheenammdath House, Rikaz Bizzari, Moza Alhammadi, Mahmoud A Hussain

**Affiliations:** 1 Department of Pediatrics, Hatta Hospital, Dubai Health, Dubai, ARE; 2 Department of Infectious Diseases, Al Jalila Children's Speciality Hospital, Dubai, ARE

**Keywords:** antibiotic stewardship, clinical management, esbl, mdr, meropenem therapy, prolonged febrile illness, typhoid fever

## Abstract

Typhoid fever, caused by *Salmonella enterica* serovar Typhi (*S. Typhi*), remains a significant global health concern. The emergence of multidrug-resistant (MDR) *S. Typhi* strains, including extended-spectrum beta-lactamase (ESBL) producers, has further complicated treatment by limiting the effectiveness of first-line and some second-line antibiotics. We present a case of a 10-year-old boy who developed a prolonged febrile illness after traveling to a typhoid-endemic region. Initial treatment with ceftriaxone proved ineffective due to the presence of ESBL-producing *S. Typhi*, necessitating a change to meropenem combined with azithromycin. Despite persistent fever, the patient showed clinical improvement by day five and became afebrile by day 11, and there was improvement of inflammatory markers and resolution of bacteremia, as confirmed by sterile blood cultures on day 12. This case underscores the challenges in managing MDR typhoid fever and highlights the critical need to maintain appropriate antibiotic regimens, even in the context of prolonged febrile responses. Furthermore, it draws attention to the global spread of MDR and extensively drug-resistant (XDR) *S. Typhi*, often facilitated by international travel, emphasizing the need for vigilant resistance monitoring and empiric treatment adjustments. Effective antibiotic stewardship, adherence to evidence-based guidelines, and heightened clinical awareness are essential to address the complex public health and clinical challenges posed by MDR and XDR *S. Typhi*.

## Introduction

Typhoid fever is a serious and sometimes life-threatening infection caused by the bacterium Salmonella enterica serovar Typhi (*S. Typhi*). It remains prevalent in many low- and middle-income countries where unsafe drinking water, poor food hygiene, and inadequate sanitation contribute to its widespread occurrence. Each year, millions of cases are reported, resulting in over 200,000 deaths globally [1]. Traditionally, typhoid fever was effectively managed with antibiotics such as chloramphenicol, ampicillin, and co-trimoxazole. However, the emergence of multidrug-resistant (MDR) strains of *S. Typhi*, which are resistant to these first-line treatments, has significantly reduced available therapeutic options. Additionally, the rise of extended-spectrum beta-lactamase (ESBL)-producing Salmonella strains has further complicated treatment by rendering antibiotics like ceftriaxone less effective [2-5]. This report highlights the clinical management of a pediatric MDR typhoid fever case complicated by ESBL production, emphasizing the challenges in antibiotic selection and the role of carbapenems in severe cases. It also underscores the expected prolonged fever in such cases and the need for strict antibiotic stewardship and monitoring of other parameters for clinical response. 

## Case presentation

Patient history and clinical features

A 10-year-old boy, a Pakistan national, residing in UAE, who recently visited a typhoid-endemic region in Pakistan, presented to Hatta Hospital on August 10, 2024, with a two-week history of high-grade intermittent fever, persistent dry cough, poor appetite, and generalized fatigue. The patient’s symptoms began shortly after returning from Pakistan. Initial evaluation at a private clinic reported Salmonella typhi H and O antibody titers of 1:160 on the Widal test, while malaria was ruled out through appropriate testing.

On examination, the patient appeared toxic and febrile, with a temperature of 39.3°C. Vital signs showed a pulse rate of 148 beats per minute and a blood pressure of 117/88 mmHg. Physical examination revealed an erythematous posterior oropharynx without associated rash, petechiae, or signs of meningeal irritation. Cardiopulmonary, abdominal, and neurological examinations were unremarkable, with no organomegaly, tenderness, or focal deficits.

Laboratory and imaging findings

**Figure 1:**
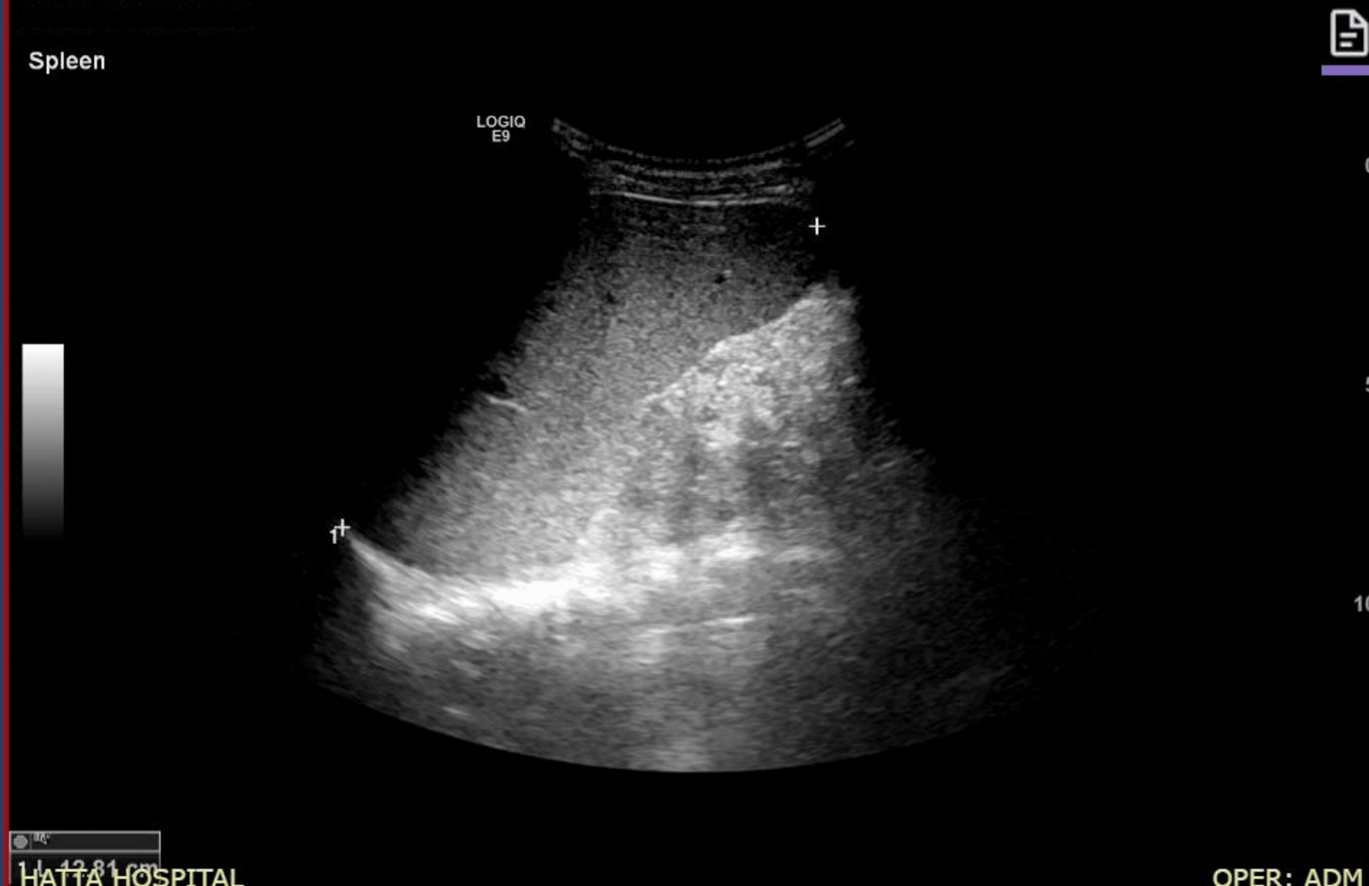
(A) Ultrasonographic Image of the Spleen The ultrasound image demonstrates an enlarged spleen, indicative of splenomegaly.

**Figure 2:**
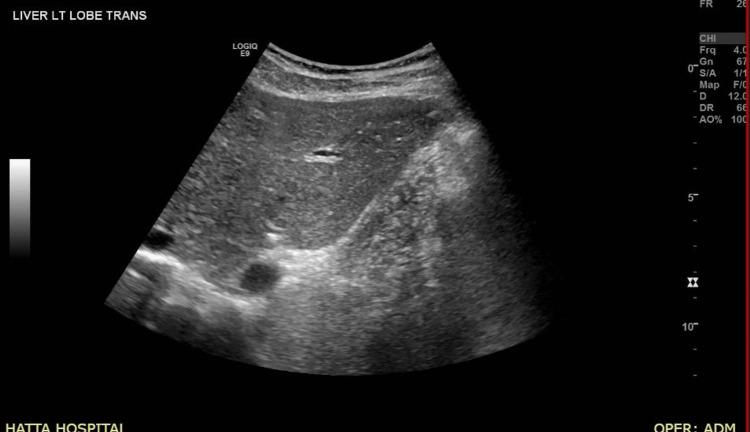
Ultrasonographic Image of the Liver (Left Lobe - Transverse View) The image reveals hepatomegaly, characterized by an enlarged liver with a mildly heterogeneous echotexture.

**Figure 3:**
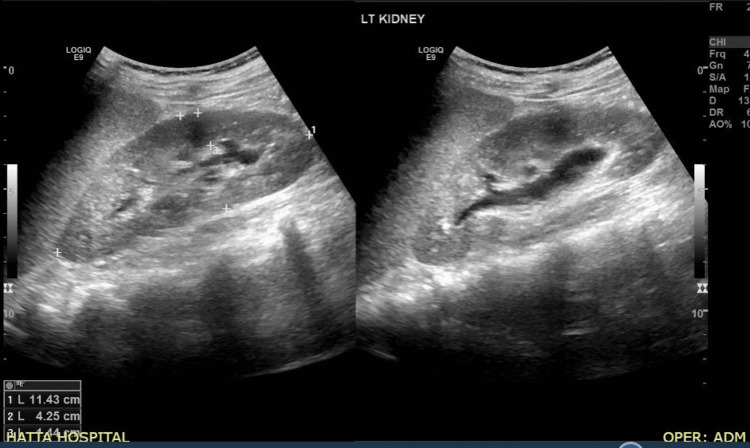
Ultrasonographic Image of the Left Kidney The ultrasound of the left kidney shows mild hydronephrosis, evidenced by slight dilation of the renal pelvis and calyces.

The initial laboratory findings indicated marked inflammation with elevated CRP (178.6 mg/L) and mild SGPT elevation, along with neutrophilia. Blood culture confirmed ESBL-producing Salmonella typhi, resistant to ceftriaxone but susceptible to meropenem and trimethoprim-sulfamethoxazole. Imaging revealed mild hydronephrosis and hepatosplenomegaly (Table 1). On follow-up (August 13, 2024), CRP declined (97.2 mg/L) but remained elevated, while SGPT and WBC normalized. Persistent neutrophilia and thrombocytosis were noted, with ongoing Salmonella typhi bacteremia (Table 2). Further follow-up (August 16, 2024) showed continued CRP reduction (75.4 mg/L), normal SGPT, and resolution of neutrophilia. Lymphocytosis was observed, and blood culture was sterile, indicating infection clearance (Table 3). Imaging demonstrated hepatosplenomegaly, mild ascites, and left hydronephrosis, with a normal echocardiogram and chest X-ray (Table 4 and Figures 1-3). Antibiotic susceptibility testing confirmed ceftriaxone resistance, with susceptibility to ertapenem, meropenem, and trimethoprim-sulfamethoxazole (Table 5).

**Table 1:** Initial Laboratory Findings CRP: C-reactive protein; SGPT (ALT): Serum glutamate pyruvate transaminase (alanine transaminase); WBC: White blood cell count; ESBL: Extended-spectrum beta-lactamase; PCR: Polymerase chain reaction; CTX-M: Cefotaximase-Munich

Parameter	Result	Normal Range	Notes
CRP	178.6 mg/L	< 5 mg/L	Markedly elevated
SGPT (ALT)	67 U/L	0-39 U/L	Mildly elevated
WBC	11 × 10³/μL	5-13 × 10³/μL	Normal
Neutrophils (%)	70.9%	40-70%	Neutrophilia
Platelets	244 × 10³/μL	170-450 × 10³/μL	Normal
Blood Culture (1st)	ESBL-producing Salmonella typhi	N/A	Resistant to ceftriaxone; sensitive to meropenem and trimethoprim-sulfamethoxazole
Blood Culture Identification (Multiplex PCR)	Salmonella spp detected, CTX-M detected	N/A	--
Imaging	Mild hydronephrosis, hepatosplenomegaly	N/A	--

**Table 2:** Follow-Up Laboratory Findings on August 13, 2024 CRP: C-reactive protein; SGPT (ALT): Serum glutamic pyruvic transaminase (alanine aminotransferase); WBC: White blood cells; ESBL: Extended-spectrum beta-lactamase

Parameter	Result	Normal Range	Notes
CRP	97.2 mg/L	< 5 mg/L	Reduced but elevated
SGPT (ALT)	45 U/L	0-39 U/L	Improved
WBC	5.6 × 10³/μL	5-13 × 10³/μL	Normal
Neutrophils (%)	77.8%	40-70%	Neutrophilia
Platelets	311 × 10³/μL	170-450 × 10³/μL	Elevated
Blood Culture (2nd)	ESBL-producing Salmonella typhi	N/A	Resistant to ceftriaxone; sensitive to meropenem and trimethoprim-sulfamethoxazole

**Table 3:** Further Follow-Up Findings on August 16, 2024 CRP: C-reactive protein; SGPT/ ALT: Serum glutamic pyruvic transaminase/alanine aminotransferase; WBC: White blood cells

Parameter	Result	Normal Range	Notes
CRP	75.4 mg/L	< 5 mg/L	Further reduced
SGPT (ALT)	45 U/L	0-39 U/L	Normal
WBC	7.4 × 10³/μL	5-13 × 10³/μL	Normal
Neutrophils (%)	42.9%	40-70%	Normal
Lymphocytes (%)	46.1%	20-40%	Elevated
Blood Culture (3rd)	Sterile	N/A	No growth

**Table 4:** Imaging Results Abdominal USS: Abdominal ultrasound scan

Imaging Type	Findings
Chest X-ray	Clear lung fields, no focal lesions, and prominent vascular markings.
Abdominal USS	Hepatomegaly, splenomegaly, mild ascites, and mild hydronephrosis (left).
Echocardiogram	No vegetation or signs of infective endocarditis.

**Table 5:** Susceptibility Profile of Salmonella typhi

Antibiotic	Susceptibility
Ceftriaxone	Resistant
Ertapenem	Susceptible
Meropenem	Susceptible
Trimethoprim-Sulfamethoxazole	Susceptible

Treatment and progress

Empirical therapy with IV ceftriaxone failed, leading to a switch to IV meropenem depending upon the blood culture report of ESBL Salmonella typhi (PCR). Early identification of organism was possible with multiplex PCR with detection of ESBL Salmonella typhi. Azithromycin was added due to concurrent detection of Mycoplasma pneumoniae IgM. Repeat blood culture after 48 hours of initiation of proper antibiotics was still positive for ESBL Salmonella typhi. Despite the initial persistence of fever, the patient showed clinical improvement by day 5 of meropenem, with improvement of inflammatory markers by day 7. He became afebrile on day 11. Follow-up blood cultures confirmed the resolution of bacteremia.

## Discussion

Typhoid fever, caused by *S. Typhi*, remains a significant global health concern, with millions of cases and a substantial mortality rate. In low- to middle-income countries, where sanitation is often inadequate, the burden of typhoid fever is particularly high, and the emergence of MDR strains has exacerbated the problem [6,7]. The rise in international travel has heightened the need for clinicians to remain alert to diseases frequently imported from other countries, as well as the resistance patterns these infections may carry [8]. Since November 2016, a significant outbreak of multidrug-resistant Salmonella Typhi has been reported in Pakistan's Sindh province, with most cases concentrated in the Hyderabad and Karachi regions [9].

Resistance to first-line antibiotics like chloramphenicol, ampicillin, and co-trimoxazole was observed as early as the 1970s, with resistance to fluoroquinolones emerging shortly after their introduction in the 1990s. As a result, third-generation cephalosporins and azithromycin have become the mainstay for treatment in regions where resistance to first-line agents is prevalent. As of December 2018, more than 5000 cases have been reported and all isolates have been resistant to ampicillin, TMP-SMX, ciprofloxacin, and ceftriaxone but remain susceptible to azithromycin [8-11].

**Figure 4:**
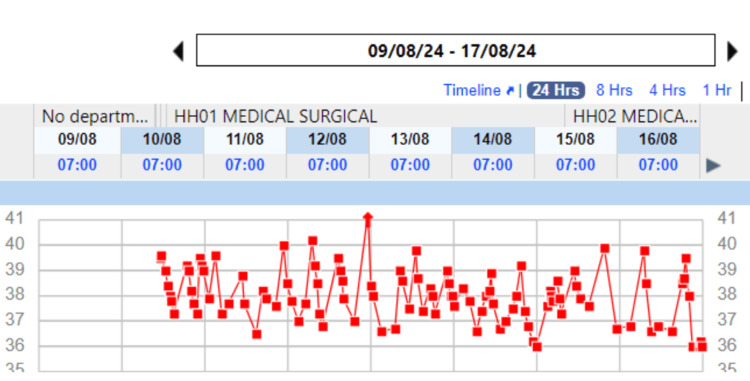
Temperature Monitoring of the Patient (August 9, 2024 - August 17, 2024) Red Squares: Recorded temperatures in degrees Celsius (°C); X-Axis: Dates of observation; Y-Axis: Temperature range in degrees Celsius (°C)

**Figure 5:**
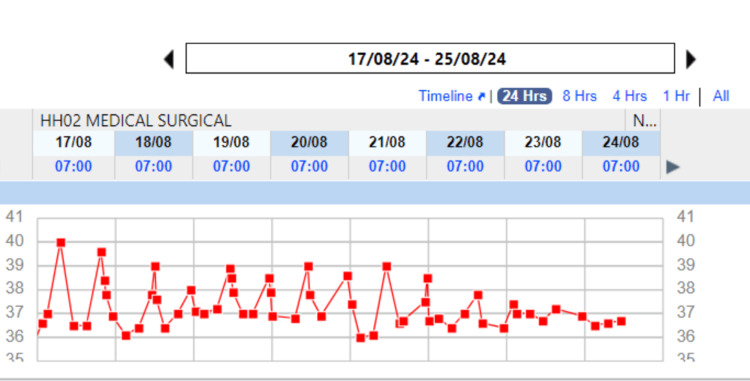
Temperature Monitoring of the Patient (August 17, 2024 - August 25, 2024) Red Squares: Recorded temperatures in degrees Celsius (°C); X-Axis: Dates of observation; Y-Axis: Temperature range in degrees Celsius (°C)

The importance of this case lies in the recognition that, despite a prolonged fever, clinicians should not hastily switch antibiotics. This case highlights the fact that fever, especially in MDR typhoid cases, may persist for longer than typically expected, and the course of the illness does not always reflect the immediate efficacy of the chosen antibiotic. The attached temperature charts (Figures 4, 5) illustrate the patient's fluctuating fever pattern over the course of treatment, emphasizing the prolonged fever typical in such cases. Clinicians should depend on other factors like improvement in symptoms and inflammatory markers. Concurrent positive mycoplasma IM titer could be a false positive finding even though we have added azithromycin considering the benefit of Azithromycin in typhoid fever.

Importantly, our patient was treated with an appropriate initial regimen of third-generation cephalosporins. This approach aligns with current treatment guidelines, which recommend ceftriaxone or cefotaxime as the first-line agents for severe or complicated cases of MDR typhoid [12].

Several studies have documented the emergence of XDR *S. Typhi* strains, particularly in Pakistan and parts of South Asia, which are resistant to multiple first-line and second-line antibiotics, including ceftriaxone and azithromycin [9-11]. The spread of such strains globally, due to increased international travel, further complicates the treatment landscape. Our case may represent an early instance of the multidrug-resistant strain outside Pakistan, which would require immediate awareness and adaptation of empiric treatment strategies to include alternative antibiotics like meropenem or a combination of ceftriaxone and azithromycin, depending on local resistance patterns.

The prolonged fever observed in this case, even after a second culture was positive, could be explained by the slow response of *S. Typhi* to treatment, particularly in MDR cases where the pathogen may exhibit delayed or partial susceptibility to certain antibiotics. It is essential for clinicians to be aware that fever may persist for several days, and this should not necessarily be interpreted as treatment failure. An echocardiogram study was done to see if any evidence of vegetation was negative. The finding of left hydronephrosis could be an incidental finding. As far as we know there is no association of typhoid fever with hydronephrosis. The decision to maintain the same antibiotic regimen, as demonstrated in our case, was appropriate and effective, as evidenced by the patient's eventual recovery.

Furthermore, this case reinforces the growing need for public health interventions aimed at preventing the spread of resistant strains of *S. Typhi*. Vaccination programs, such as the Typbar-TCV® vaccine, have shown promise in reducing the incidence of typhoid fever in endemic regions. The World Health Organization (WHO) has endorsed large-scale vaccination efforts, particularly in areas with high endemicity, to combat the rise of resistant strains and reduce the burden of the disease [13,14].

## Conclusions

The management of MDR typhoid fever remains challenging due to the evolving resistance patterns of *S. Typhi*. This case underscores the importance of not prematurely altering antibiotic regimens in the face of prolonged fever, especially when cultures are intermittently negative. Clinicians should be vigilant about the presence of resistant strains and the impact of travel-associated infections on local health systems. Continued surveillance, antibiotic stewardship, and vaccination efforts are essential to control the spread of MDR and XDR typhoid fever globally.
